# Efficient Expression of Genes in the *Drosophila* Germline Using a UAS Promoter Free of Interference by Hsp70 piRNAs

**DOI:** 10.1534/genetics.118.300874

**Published:** 2018-04-18

**Authors:** Steven Z. DeLuca, Allan C. Spradling

**Affiliations:** Howard Hughes Medical Institute Research Laboratories, Department of Embryology, Carnegie Institution for Science, Baltimore, Maryland 21218

**Keywords:** UASt promoter, UASp promoter, Hsp70, piRNAs, *Drosophila*, female, germ cell

## Abstract

Controlling the expression of genes using a binary system involving the yeast GAL4 transcription factor has been a mainstay of *Drosophila* developmental genetics for nearly 30 years. However, most existing GAL4 expression constructs only function effectively in somatic cells, but not in germ cells during oogenesis, for unknown reasons. A special upstream activation sequence (UAS) promoter, UASp was created that does express during oogenesis, but the need to use different constructs for somatic and female germline cells has remained a significant technical limitation. Here, we show that the expression problem of UASt and many other *Drosophila* molecular tools in germline cells is caused by their core *Hsp70* promoter sequences, which are targeted in female germ cells by *Hsp70*-directed Piwi-interacting RNAs (piRNAs) generated from endogenous *Hsp70* gene sequences. In a genetic background lacking genomic *Hsp70* genes and associated piRNAs, UASt-based constructs function effectively during oogenesis. By reducing *Hsp70* sequences targeted by piRNAs, we created UASz, which functions better than UASp in the germline and like UASt in somatic cells.

## Abstract (precis)

Using the yeast GAL4 transcription factor to control expression in *Drosophila melanogaster* has long been ineffective in female germ cells during oogenesis. Here, DeLuca and Spradling show that the expression problem of most *Drosophila* molecular tools...

*DROSOPHILA* is an extremely powerful model organism for studies of animal development and disease because of its low maintenance costs, rapid generation time, and expansive collection of tools to genetically modify its cells. One particularly useful tool is the Gal4/upstream activation sequence (UAS) two-component activation system, in which the Gal4 transcriptional activator protein recognizes a UAS to induce the expression of any gene of interest (Fischer *et al.* 1988; Brand and Perrimon 1993). By controlling the activity of Gal4 with tissue-specific or inducible promoters, or the Gal80 inhibitor protein, one can manipulate genes in specific cells or times of development, visualize cell types, probe cell function, or follow cell lineages. One of the most useful applications of these techniques has been to carry out genetic screens by expressing RNA interference (RNAi) in targeted tissues or cultured cells (Dietzl *et al.* 2007; Ni *et al.* 2008).

The original pUASt vector from Brand and Perrimon (1993), which contains an *Hsp70*-derived core promoter and simian virus 40 terminator, has undergone several optimizations to improve its expression (Figure 1A). Popular versions, such as the Valium10 or 20 vector used by the *Drosophila* Transgenic RNAi project (TRiP) (Ni *et al.* 2009, 2011) and the pMF3 vector used by the Vienna *Drosophila* Research Center (VDRC) GD collection (Dietzl *et al.* 2007) added a ftz intron, and the Janelia Gal4 enhancer project used derivatives of pJFRC81, which added a myosin IV intron (IVS), synthetic 5′-UTR sequence (syn21), and viral p10 terminator to boost expression levels across all *Drosophila* cell types (Figure 1A) (Pfeiffer *et al.* 2012). However, these modifications did not correct UASt’s major problem, that it drives woefully poor expression in the female germline compared to somatic tissues. Consequently, genetic manipulation in this important tissue has often relied on a special GAL4-activated promoter, UASp, produced by fusing 17 copies of the UAS activator to a germline-compatible promoter derived from the *P*-element, a transposon naturally active in the female germline (Figure 1B) (Rørth 1998). Although UASp expression is qualitatively higher than UASt in the female germline, it is generally known to be lower in somatic tissues.

**Figure 1:**
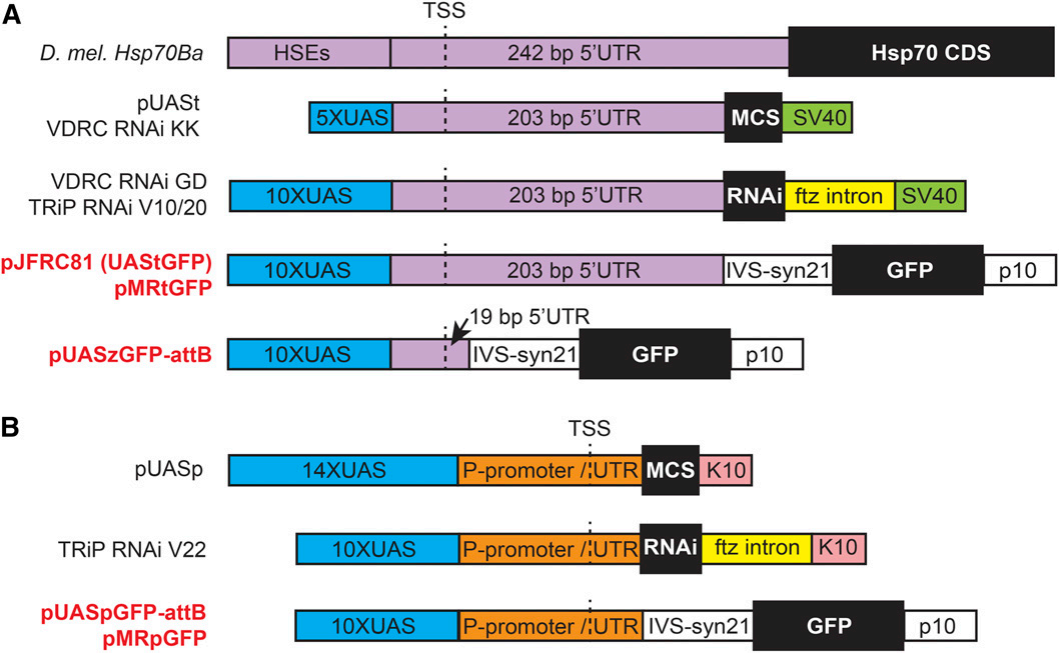
Components of common upstream activator sequence (UAS) constructs used by the fly community. (A) Cartoon depicting a *Drosophila* *Hsp70* gene relative to sequences in UASt-based vectors. In pUASt, multiple copies of optimized Gal4-binding sites (5xUAS) replace heat-inducible enhancers (Heat Shock Elements, HSEs) in a fragment of Hsp70 containing the transcription start site (TSS) and 5′-UTR. In derivatives of UASt, a multiple cloning site (MCS), RNA interference (RNAi) constructs, GFP coding sequence, synthetic UTR elements (syn21), and introns (ftz or myosin IV, IVS) replace 39 bp of the Hsp70 5′-UTR and Hsp70 coding sequence (CDS). Viral-derived simian virus 40 (SV40) or p10 sequences terminate transcription and contribute to the 3′-UTR. For this study, we created a derivative of pJFRC81 with a truncated 5′-UTR (pUASzGFP-attB) and a derivative compatible with MiMIC recombinase-mediated cassette exchange (pMRtGFP). (B) Cartoon depicting two common UASp vectors containing the K10 terminator and *Drosophila* P-element promoter, TSS, and 5′-UTR in place of the SV40 terminator and Hsp70 sequences. We created two new UASp vectors, pUASpGFPattB and pMRpGFP, based on pJFRC81and pMRtGFP, to directly compare the effect of P-element and Hsp70 sequences on transgene expression. Vector names colored red are used in this study. TRiP, *Drosophila* Transgenic RNAi project; VDRC, Vienna *Drosophila* Research Center.

The lack of a UAS construct that is widely useful in all *Drosophila* tissues has remained an obstacle to providing optimum genetic tools to the research community. Transgenic RNAi collections were first constructed using UASt, and screening of genes for germline functions has relied on increasing the effectiveness of RNAi by coexpressing Dcr2 or expressing short hairpin RNAi from UASp promoters (Ni *et al.* 2011; Yan *et al.* 2014; Sanchez *et al.* 2016). A significant obstacle to obtaining a widely effective GAL4 vector has been the lack of understanding of the reason that UASt functions poorly in germ cells, and the paucity of accurate comparisons between the UASp and UASt promoters in the absence of other significant variables.

## Materials and Methods

### *Drosophila* strains

Mef2-Gal4 (BL26882) w[*]; Kr[If-1]/CyO, P{w+ GAL4-Mef2.R}2, P{w+ UAS-mCD8.mRFP}2.
Tub-Gal4 (BL5138) y[1] w[*]; P{w+ tubP-GAL4}LL7/TM3, Sb[1] Ser[1].
FLP/φC31int (BL33216) P{hsFLP}12, y[1] w[*] M{vas-int.B}ZH-2A; S[1]/CyO; Pri[1]/TM6B, Tb[1].
Hsp70∆ (BL8841) w[1118]; Df(3R)Hsp70A, Df(3R)Hsp70B.
Nanos-Gal4VP16 (BL4937) w[1118] ;; P{nos-GAL4::VP16-nos.UTR}^MVD1^.
*Vasa-Gal4* was obtained from Zhao Zhang’s laboratory: *y*[*] *w*[*];; *P*{*w+ vas-GAL4.2.6*} (Zhao *et al.* 2013).
UASt-Rhi-RNAi (VDRC313156 from Julius Brennecke’s stocks).
UASt-w-RNAi (VDRC313772 from Julius Brennecke’s stocks).

### New stocks created for this study

Bestgene introduced pMRtGFP and pMRpGFP into yw flies using a P-transposase helper plasmid, and we isolated GFP+ insertions by crossing the F0 to a *Mef2-Gal4* background and scoring for GFP+ muscles. We introduced UAStGFP or UASpGFP into MI04106 and other MiMIC lines using a cross strategy outlined in Nagarkar-Jaiswal *et al.* (2015). Rainbow transgenics introduced pJFRC81 (UAStGFP-attB), pUASpGFP-attB, and pUASzGFP-attB into attP40 using an X chromosome-encoded φC31 integrase source, and we isolated multiple w^+^, φC31 minus insert lines by standard fly genetics.

### Vectors created for this study

Genescript synthesized pMRtGFP. We created pMRpGFP by replacing the *Nhe*I-*Bgl*II UASt promoter in pMRtGFP with a *Spe*I-*Bgl*II UASp promoter from Valium22. We created pUASpGFP-attB by replacing the *Pst*I-*Bgl*II UASt promoter in pJFRC81 with the *Pst*I-*Bgl*II UASp promoter from Valium22. We created UASzGFP-attB by replacing the 259-bp *Nhe*I-*Bgl*II fragment of pJFRC81 containing the 203-bp *Hsp70* promoter with annealed oligos encoding 63 bp from the 5′ end of the same promoter.

Top oligo: 5′-CTAGCGACGTCGAGCGCCGGAGTATAAATAGAGGCGCTTCGTCTACGGAGCGACAATTCAATTCAAACAAGCAAA-3′.
Bottom oligo: 5′-GATCTTTGCTTGTTTGAATTGAATTGTCGCTCCGTAGACGAAGCGCCTCTATTTATACTCCGGCGCTCGACGTCG-3′.

We created UASz by replacing the *Not*I-syn21-GFP-*Xba*I fragment in UASzGFP with annealed oligos encoding *Not*I-sny21-*Bam*HI-*Xho*I-*Kpn*I-*Spe*I-*Xba*I.

Top oligo: 5′-GGCCGCAACTTAAAAAAAAAAATCAAAGGATCCCTCGAGGGTACCACTAGTT-3′.
Bottom oligo: 5′-CTAGAACTAGTGGTACCCTCGAGGGATCCTTTGATTTTTTTTTTTAAGTTGC-3′.

We created UASz1.1 by replacing the *Kpn*I-*Eco*RI p10 terminator in UASz with a PCR-amplified p10 terminator containing Kpn1-*Xba*I-*Eco*RI and ApoI tails.

Forward primer: 5′-CATGGTACCGCCTCTCTAGAGTGTGAATTCTGGCATGAATCGTTTTTAAAATAACAAATCAATTGTTTTATAAT-3′.
Revers primer: 5′-GGAAATTTTCGAATCGCTATCCAAGCCAGCT-3′.

We created UASz1.2 by destroying the *Nhe*I and *Eco*RI sites in UASz1.1 by cloning annealed oligos into the *Nhe*I-*Eco*RI backbone.

Top oligo: 5′-CTAGGAGCGCCGGAGTATAAATAGAGGCGCTTCGTCTACGGAGCGACAATTCAATTCAAACAAGCAAGATCTGGCCTCGAGT-3′.
Bottom oligo: 5′-AATTACTCGAGGCCAGATCTTGCTTGTTTGAATTGAATTGTCGCTCCGTAGACGAAGCGCCTCTATTTATACTCCGGCGCTC-3′.

To create UASzMiR, we cloned a *Bgl*II-*Xho*I fragment containing the MiR1 cassette and ftz intron from Walium22 into the *Bgl*II-*Xho*I backbone of UASz1.2.

### Tissue preparation, imaging, and quantitation

**Figure 2:**
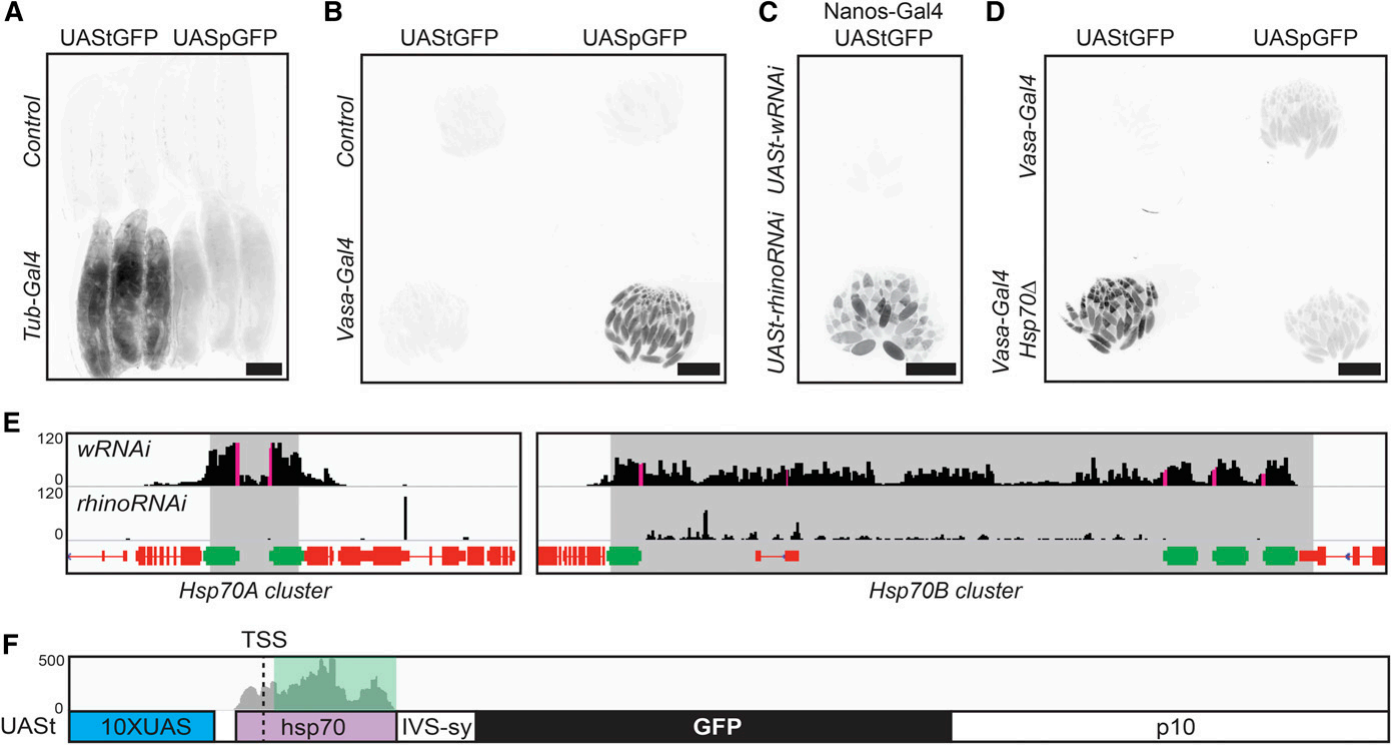
Expression from UASt is greater than UASp in cells lacking *Hsp70* piRNAs. (A–D) pMRtGFP (UAStGFP) and pMRpGFP (UASpGFP) integrated into the same MiMIC site (Mi04106) and crossed to either a control without Gal4 to visualize UAS-GFP leakiness, *Tub-Gal4* for somatic UAS-GFP expression, or *Vasa-Gal4* or *Nanos-Gal4* for germline UAS-GFP expression. Each panel is a single inverted GFP fluorescence image, genotypes mounted side-by-side to compare expression levels. Scale bar is 1 mm. (A) Wandering third-instar larvae. (B–D) Adult ovaries. (C) Nanos-Gal4 driving germline UAStGFP expression in addition to w-RNAi (control) or Rhino-RNAi. (D) Germline UAS-GFP expression in the presence or absence of *Hsp70* genes and piRNAs. Image in (B) is longer exposure than (D) to show minimal induction of UAStGFP when vasa-Gal4 is added in the presence of *Hsp70* piRNAs. (E and F) Genome browser view of whole-ovary-derived piRNAs from MTD-Gal4 > UASt-wRNAi (control) or MTD-Gal4 > UASt-rhinoRNAi (Mohn *et al.* 2014) aligned to *Drosophila* genome release 6 (E) or our UAStGFP construct (F). (E) *Hsp70* genes colored in green. piRNA read depth in black. piRNA read depth also mapping to *UASt* in magenta. Gray shaded area represents the DNA deleted in the *Hsp70*∆ background. (F) piRNAs from MTD-Gal4 > UASt-wRNAi aligned to our UAStGFP construct to show the mapping position of previously described piRNAs that could theoretically silence the transgenes used in this study. The green shaded area shows the 184 bp of UASt deleted to make UASz. piRNA, Piwi-interacting RNAs; RNAi, RNA interference; UAS, upstream activator sequence.

For all experiments, we crossed UAS-GFP or UAS-GFP *Hsp70*∆ males to control (*yw*), *Tub-Gal4/TM3*, homozygous *Vasa-Gal4*, or homozygous *Vasa-Gal4 Hsp70*∆ females. For whole-larvae imaging, we picked wandering third-instar larvae of various genotypes, aligned them on the same glass slide, and placed them the freezer for 30 min prior to imaging. For adult ovary or larval tissue imaging, we fixed dissected tissue with 4% paraformaldehyde for 13 min (whole ovary) or 20 min (larval tissue) and stained with DAPI in PBS with 0.1% Triton X-100. We imaged the GFP fluorescence of semifrozen whole third-instar larvae or whole ovaries mounted in 50% glycerol on a Leica Stereoscope equipped with a mercury arc light source, GFP filters, and a CCD camera. We imaged GFP fluorescence in larval imaginal discs, salivary glands, and epidermis, and manually separated ovarioles mounted in 50% glycerol using a custom-built spinning disc confocal with a 20× 0.8 NA lens. For each genotype and tissue type, we acquired a single-plane image from at least four individuals using Metamorph software and the same laser power, CCD camera gain, and exposure time between equivalent samples. We measured average pixel intensity in 14-bit images of the GFP channel using Image J. We acquired representative images of single planes through single ovarioles for Figure 2 on a Leica Sp8 scanning confocal with a 63× 1.4 NA lens and PMT (for DAPI) and HiD (GFP) detectors using identical settings between samples.

For UASt Piwi-interacting RNAs (piRNA) analysis, we clipped and aligned sequenced small RNA libraries from Mohn *et al.* (2014) (SRR1187947:control germline knockdown and SRR1187948:rhino germline knockdown) to the *Drosophila melanogaster* genome Release 6 (Hoskins *et al.* 2015) or UAStGFP using the Bowtie2 aligner with no filtering for repetitive mappers (Langmead and Salzberg 2012). We visualized piRNA read depth to UAStGFP or both Hsp70 clusters using the Interactive Genome Browser (Robinson *et al.* 2011).

### Data availability

Fly strains and vectors are available upon request. pUASz1.0 and pUASzMiR, and sequences, are available from the *Drosophila* Genomics Resource Center as items 1431 and 1432. Supplemental material available at Figshare: https://doi.org/10.25386/genetics.6089828.

## Results and Discussion

### Difference between UASp and UASt

To study the difference between the UASp and UASt promoters, we first created UAStGFP and UASpGFP constructs controlled for other variables between the original UASt and UASp, such as UTR components, introns, terminators, and genomic insertion site. Both constructs were based on pJFRC81 and only varied at the promoter and 5′-UTR of the transcript (Figure 1, red letters). We made pMRtGFP and pMRpGFP compatible with φC31-catalyzed recombination-mediated cassette exchange with MiMIC transposons, allowing us to integrate UAS-GFPs into many common sites throughout the genome (Venken *et al.* 2011). Using a previously established protocol (Nagarkar-Jaiswal *et al.* 2015), we recombined both UAS-GFPs into several MiMICS, including MI04106, which resides in a region enriched for ubiquitously expressed genes and active chromatin marks (Filion *et al.* 2010; Kharchenko *et al.* 2011), referred to as “the gooseneck” by Calvin Bridges for its long stretch of low density in salivary gland polytene chromosome preparations (Bridges 1935). Consistent with previous reports, UASt drove significantly stronger expression than UASp in all somatic tissues examined, while UASp drove significantly stronger expression in the female germline (Figure 2, A and B).

### Hsp70 piRNAs repress UASt

We next investigated the reason for the extremely weak UASt expression in the female germline. Several lines of evidence implicated piRNA-directed silencing as a mechanism limiting UASt expression. *Drosophila* piRNAs are ovary -and testis-enriched, 23–29-nt RNAs that complex with Argonaut family proteins and silence transposons through homologous base pairing-directed mRNA cleavage and heterochromatin formation (Siomi *et al.* 2011). Some of the most successful UASt-based genetic screens in the female germline knocked down piRNA biogenesis genes (Czech *et al.* 2013; Handler *et al.* 2013). If piRNAs were silencing UASt, then UASt-RNAi against a piRNA biogenesis gene would boost UASt expression leading to maximal knockdown. Where might these UASt-piRNAs originate from? Previously, Olovnikov *et al.* (2013) characterized an abundance of germline-specific piRNAs mapping to both *Hsp70* gene clusters. Because UASt contains the *Hsp70* promoter and 5′-UTR, we hypothesized that germline piRNAs against *Hsp70* may be targeting UASt. When we searched for UASt sequences in the piRNAs identified by Mohn *et al.* (2015), we identified abundant piRNAs perfectly homologous to UASt (Figure 2E, pink bars, and Figure 2F). Similar to UASt silencing, these UASt piRNAs are restricted to the female germline because germline-specific knockdown of *rhino*, a gene required for Hsp70 piRNA production, eliminates UASt piRNAs from whole ovaries (Figure 2E) (Mohn *et al.* 2014).

To directly test whether Hsp70 piRNAs silence UASt, we tested UASt expression in *Hsp70*∆ flies (Gong and Golic 2004), which completely lack all genetic loci producing piRNAs homologous to UASt (Figure 2E, gray boxes deleted). Despite missing all copies of the inducible *Hsp70* gene family and related piRNAs, *Hsp70*∆ flies have no significant defects in viability or egg production in the absence of heat stress (Gong and Golic 2006). However, *Hsp70*∆ flies showed greatly enhanced UAStGFP expression. Furthermore, UAStGFP expression was significantly stronger than that of UASpGFP, which was unaffected by *Hsp70*∆ (Figure 2D). Repression of full UAStGFP expression in germ cells requires normal piRNA production, since UAStGFP expression was also boosted by germline knockdown of *rhino*, which is required for the production of Hsp70 and many other germline-specific piRNAs (Figure 2C). These results argue strongly that UASt is normally silenced by *Hsp70* piRNAs and that UASt is a stronger expression vector than UASp in cells lacking *Hsp70* piRNAs.

### Construction of UASz

We next attempted to create a new version of the UAS expression vector that works well in both the soma and the female germline. We hypothesized that eliminating the part of UASt that is targeted by piRNAs would boost UASt expression by the same amount as eliminating the piRNAs themselves. *Hsp70* piRNAs are homologous to 247 nt of the UASt promoter and 5′-UTR. While we could make enough substitutions along this stretch to prevent all possible 23-nt piRNAs from binding, we were afraid that this approach might impair important promoter sequences. Instead, we hypothesized that *Hsp70* piRNAs might recognize UASt RNA to initiate piRNA silencing. To prevent *Hsp70* piRNAs from recognizing UASt RNA, we trimmed down the UASt 5′-UTR to be shorter than a single piRNA, from 213 to 19 nt (Figure 1A and Figure 2E). We named this UTR-shortened UASt variant “UASz,” because we optimistically hoped that it would be the last one anyone would make.

### Comparison of UAS vectors

**Figure 3:**
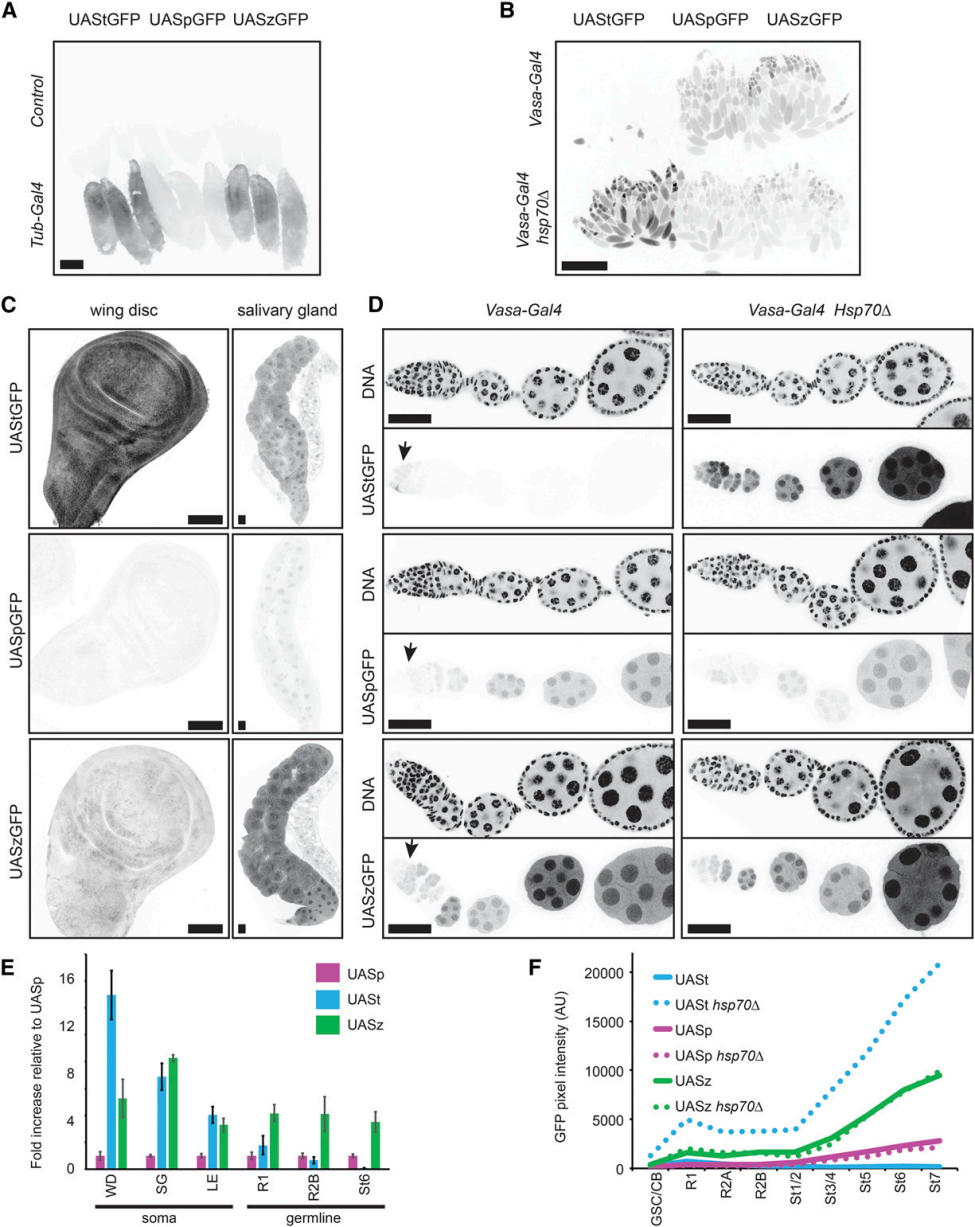
Expression level of *UASz* relative to current *UAS* variants. UAStGFP, UASpGFP, and UASzGFP integrated into a single genomic site, attP40, crossed to control (no *Gal4*) or the indicated *Gal4* driver in either a wild-type or *Hsp70*∆ background. Inverted GFP fluorescence images of wandering third-instar larvae (A), whole adult ovaries (B), or third-instar larval WDs and SGs (C). (D) Paired images showing single ovarioles of the indicated genotype imaged in one channel for GFP fluorescence (bottom) and another for DAPI (DNA, top). Arrows indicate germline region 1, where piRNA silencing is reduced. Scale bars are 1 mm for (A and B) and 0.1 mm for (C and D). (E) Average GFP fluorescence intensity from UAStGFP and UASzGFP relative to UASpGFP in the indicated tissue expressing *Tub-Gal4* (soma) or *Vasa-gal4* (germline). Error bars indicate SD from the mean from at least four samples. (F) Average GFP pixel intensity in germ cells of the indicated stage and genotype. R1, R2A, or R2B indicates germline region 1, 2A, or 2B. GSC/CB, germline stem cell or cystoblast; LE, larval epidermis; Piwi-interacting RNAs; SG, salivary gland; St_, nurse cells of indicated stage number; UAS, upstream activator sequence; WD, wing disc.

To compare the relative expression levels of our UASz to UASp and UASt, we created all three variants in the same GFP vector backbone (pJFRC81) with a single attB site. We used φC31 integrase to introduce these UAS-GFP variants into a commonly used genomic site, attP40, and recombined all three inserts with *Hsp70*∆ to determine the influence of *Hsp70* piRNAs on their expression. When combined with *Tub-Gal4*, a somatic Gal4 driver, UASz was expressed at least four times higher than UASp in all somatic tissues tested and was equivalent or greater than UASt in some somatic tissues, like the larval epidermis and salivary gland (Figure 3, A, C, and E). However, UASz was expressed at ∼40% of UASt in discs, suggesting that some elements of the UASt 5′-UTR may boost expression in some tissues (Figure 3, C and E). To measure germline expression, we crossed the three *UAS-GFPs* to *vasa-Gal4*, which is evenly expressed up to stage six of oogenesis. In the germline, UASz was expressed around four times higher than UASp at all stages, while UASt was expressed at much lower levels than UASp, except in region 1 of the germarium (Figure 3, B and D–F), where piRNA silencing is weaker (Dufourt *et al.* 2014). We conclude that UASz is a superior expression vector to UASp in all tissues, and is equivalent to UASt in many, but not all, somatic tissues.

Finally, we wanted to test if UASz is still targeted by Hsp70 piRNAs because it contains 63 nt of Hsp70 sequence and ∼10% of the putative piRNAs targeting UASt (Figure 2F). We crossed UASzGFP into the *Hsp70*∆ background and compared UASzGFP levels with or without Hsp70 piRNAs. We observed no enhancement of UASzGFP when Hsp70 piRNAs were removed (Figure 3, B, D, and F). Therefore, Hsp70 piRNAs likely target the UASt but not the UASz 5′-UTR, consistent with the model that piRNAs must initially recognize RNA but not DNA.

Is UASz the final, fully optimized iteration of a UAS vector? Probably not. UASt without Hsp70 piRNAs induces about twice the expression of UASz in the ovary (Figure 3, B, D, and F). This twofold advantage of UASt over UASz in the germline or imaginal discs lacking Hsp70 piRNAs is similar to the twofold advantage of UASt over the UAS fused to the *Drosophila* Synthetic Core Promoter (Pfeiffer *et al.* 2010). Perhaps adding back some sequences within the first 203 nt of the Hsp70 5′-UTR while avoiding piRNA recognition may improve UASz. However, the current iteration of UASz remains an unequivocal upgrade over UASp for all applications and UASz should be preferred over UASt if both germline and soma studies are planned from a single vector. Alternatively, one could boost germline expression of an existing UASt construct by crossing it into the *Hsp70*∆ background.

Current UAS-RNAi collections are heavily biased toward UASt-RNAi-based constructs. To date, the VDRC and DRSC/TRiP RNAi projects have used UASt-RNAi to target 12,539 and 8876 genes, respectively. Germline screens for developmental phenotypes using UASt-RNAi were enriched for phenotypes in germline region 1 (Yan *et al.* 2014; Sanchez *et al.* 2016), where piRNA silencing is weakest (Dufourt *et al.* 2014), and UASt shows maximum expression (Figure 3D arrow). Perhaps these screens were depleted for developmental defects in later germline stages because of poor UAS-RNAi expression in these stages. Although UASp-RNAi from the Valium22 vector (Figure 1B) increased the efficiency of obtaining phenotypes in a germline screen, only 1596 genes are currently targeted by this collection (Yan *et al.* 2014). Additionally, when screening somatic cells, Ni *et al.* (2011) recommend UASt-RNAi because UASp-RNAi gave incomplete knockdowns. Our results revealed that UASp is equally weak in the germline as somatic tissues when compared to UASz (Figure 3E). Therefore, UASp-RNAi may also generate incomplete knockdowns in the germline. To increase germline RNAi expression, we propose using a UASz-based RNAi expression vector, such as UASzMiR (Supplemental Material, Figure S1), which is compatible with previously generated short hairpin RNA oligo cloning (Ni *et al.* 2011).
